# Asymptomatic Primary Infection with Epstein-Barr Virus: Observations on Young Adult Cases

**DOI:** 10.1128/JVI.00382-17

**Published:** 2017-10-13

**Authors:** Rachel J. Abbott, Annette Pachnio, Isabela Pedroza-Pacheco, Alison M. Leese, Jusnara Begum, Heather M. Long, Debbie Croom-Carter, Andrea Stacey, Paul A. H. Moss, Andrew D. Hislop, Persephone Borrow, Alan B. Rickinson, Andrew I. Bell

**Affiliations:** a Institute of Immunology and Immunotherapy, University of Birmingham, Birmingham, United Kingdom; b Institute of Cancer and Genomic Sciences, University of Birmingham, Birmingham, United Kingdom; c Nuffield Department of Clinical Medicine, University of Oxford, Oxford, United Kingdom; Northwestern University

**Keywords:** CD8 T cell, Epstein-Barr virus, host immune response, infectious mononucleosis, NK cell, primary infection

## Abstract

Epstein-Barr virus (EBV) is typically acquired asymptomatically in childhood. In contrast, infection later in life often leads to infectious mononucleosis (IM), a febrile illness characterized by anti-EBV IgM antibody positivity, high loads of circulating latently infected B cells, and a marked lymphocytosis caused by hyperexpansion of EBV-specific CD8^+^ T cells plus a milder expansion of CD56^dim^ NKG2A^+^ KIR^−^ natural killer (NK) cells. How the two situations compare is unclear due to the paucity of studies on clinically silent infection. Here we describe five prospectively studied patients with asymptomatic infections identified in a seroepidemiologic survey of university entrants. In each case, the key blood sample had high cell-associated viral loads without a marked CD8 lymphocytosis or NK cell disturbance like those seen in patients during the acute phase of IM. Two of the cases with the highest viral loads showed a coincident expansion of activated EBV-specific CD8^+^ T cells, but overall CD8^+^ T cell numbers were either unaffected or only mildly increased. Two cases with slightly lower loads, in whom serology suggests the infection may have been caught earlier in the course of infection, also showed no T or NK cell expansion at the time. Interestingly, in another case with a higher viral load, in which T and NK cell responses were undetectable in the primary blood sample in which infection was detected, EBV-specific T cell responses did not appear until several months later, by which time the viral loads in the blood had already fallen. Thus, some patients with asymptomatic primary infections have very high circulating viral loads similar to those in patients during the acute phase of IM and a cell-mediated immune response that is qualitatively similar to that in IM patients but of a lower magnitude. However, other patients may have quite different immune responses that ultimately could reveal novel mechanisms of host control.

**IMPORTANCE** Epstein-Barr virus (EBV) is transmitted orally, replicates in the throat, and then invades the B lymphocyte pool through a growth-transforming latent infection. While primary infection in childhood is usually asymptomatic, delayed infection is associated with infectious mononucleosis (IM), a febrile illness in which patients have high circulating viral loads and an exaggerated virus-induced immune response involving both CD8^+^ T cells and natural killer (NK) cells. Here we show that in five cases of asymptomatic infection, viral loads in the blood were as high as those in patients during the acute phase of IM, whereas the cell-mediated responses, even when they resembled those in patients during the acute phase of IM in timing and quality, were never as exaggerated. We infer that IM symptoms arise as a consequence not of the virus infection *per se* but of the hyperactivated immune response. Interestingly, there were idiosyncratic differences among asymptomatic cases in the relationship between the viral load and the response kinetics, emphasizing how much there is still to learn about primary EBV infection.

## INTRODUCTION

Epstein-Barr virus (EBV), a gammaherpesvirus-1 with B cell growth-transforming ability, is widespread in all human populations and is carried by most individuals as a seemingly harmless infection (1). The virus is orally spread and is typically acquired from close family contacts during infancy or early childhood, at which time infection is almost always clinically silent. However, with rising standards of hygiene in the developed world, primary infection is increasingly being delayed until the second or third decade of life. This often leads to infectious mononucleosis (IM), an illness whose symptoms (fever, sore throat, cervical lymphadenopathy, and fatigue) range from mild to severe and may take between 1 and 6 weeks to resolve (2). The proportion of delayed primary infections that are symptomatic varies from 25 to >80% in different reports, likely reflecting differences in the rigor of follow-up and clinical monitoring (3–6). Such symptomatic cases have been extensively studied; indeed, what little is known about the virology and immunology of primary EBV infection comes almost exclusively from IM patients. Thus, by the time that IM symptoms appear (typically, 5 to 7 weeks after oral transmission), high levels of infectious virus can be detected in throat washings, reflecting lytic virus replication in epithelial cells and probably also B cells at oropharyngeal surfaces (7, 8). Also, by this time, the virus has already spread into the general B cell pool as a latent infection. Many latently infected B cells can be found both in tonsillar lymphoid tissue, where proliferating EBV growth-transformed cells exist alongside cells with various degrees of more restricted latency (9, 10), and in the blood, where most, if not all, cells have fully suppressed EBV growth-transforming protein expression, moved to a resting state, and entered the recirculating memory B cell pool (11). While virus shedding in the throat may continue at a high level for several months after IM symptoms have waned (5, 12), the number of latently infected cells in the blood tends to fall quickly during the disease course itself and then falls more gradually to reach a stable set point over the following weeks and months (11, 13).

These events are coincident with a range of immune responses (14). Thus, IM can be diagnosed serologically through the presence of IgM and rising titers of IgG antibodies to the lytic EBV viral capsid antigen (VCA) in the absence of an IgG response to the latent nuclear antigen EBNA1; thereafter, the IgM response gradually subsides, the anti-VCA IgG titer stabilizes, and, months later, the anti-EBNA1 IgG response appears (2). Blood collected from patients during the acute phase of IM is also characterized by a large expansion of CD8^+^ T cells with an activated effector (CD38^+^ HLA-DR^+^ CCR7^−^ CD45RA^−^), apoptosis-prone (Bcl2^low^) phenotype. Many of these activated cells are specific for immunodominant EBV antigens of the immediate early and early lytic cycle, and these are accompanied by smaller (sometimes delayed) responses against latent antigens (15, 16). The CD8 cell expansion in the blood is most marked in patients with clinically more severe cases of IM (5) and contracts slowly as symptoms resolve, generating a memory population of resting EBV-specific cells. Some memory cells reexpress the CCR7 lymphoid homing marker, facilitating entry into tonsillar tissue and, ultimately, the establishment of a tissue-resident memory population at this site (17, 18). There is also a comparatively weak (median, <2-fold) expansion of circulating natural killer (NK) cell numbers in patients during the acute phase of IM, reflecting an increase in cells whose phenotype (CD56^dim^ NKG2A^+^ KIR^−^) is intermediate between the phenotypes of the less mature (CD56^bright^ NKG2A^+^ KIR^−^) and more mature (CD56^dim^ NKG2A^−^ KIR^+^) subsets of NK cells present in blood (19–21). At the same time, circulating dendritic cells of the myeloid (mDC) and, especially, the plasmacytoid (pDC) subsets are reduced in number (20, 22). Finally, the levels of a range of antiviral and/or proinflammatory cytokines and chemokines are reported to be elevated in the plasma of IM patients, including beta interferon (IFN-β), IFN-γ, interleukin-1 (IL-1), IL-6, IL-10, IL-12, IL-18, tumor necrosis factor alpha (TNF-α), monokine induced by IFN-γ (MIG; CXCL9), and IFN-γ-induced protein 10 (IP-10; CXCL10) (23–26). Since many of these cytokines and chemokines can be produced by multiple different cell types, it is difficult to say whether they derive from infected cells *per se* or from cells activated as part of the immune response to infection.

The factors determining whether primary EBV infection is asymptomatic or presents as IM are poorly understood. Clearly, the age at which the virus is acquired is important. In that context, the greater risk of IM among adolescents and young adults than among children has been variously ascribed to their greater chance of acquiring a high initial virus dose by kissing (14), to the diminishing competence with age of early NK cell control over new virus acquisition (19), and to the increasing breadth with age of T cell memory, such that responses to a new agent may be inflated by cross-reactive recognition from previously primed specificities (27). That said, the effect of age is not absolute because classical IM is occasionally seen in pediatric cohorts (13, 19) and may indeed be underrecognized there. Furthermore, epidemiologic studies have found a greater concordance of the incidence of IM among monozygotic twins than among dizygotic twins and first-degree relatives, strongly implying a genetic element to the risk of IM that is superimposed upon environmental influences (28, 29).

A major barrier to progress in this field is our almost complete ignorance of the virologic and immunologic events that occur in asymptomatic primary infection. Some early studies attempted to address these issues in pediatric cohorts but were largely limited to serologic screening or to the limited cellular immunologic assays available at that time (30–32). Several more recent reports have monitored EBV acquisition in African children but mainly in circumstances not only in which it was difficult to assess symptomatology but also in which confounding factors affecting immune competence, notably, coinfection with HIV and/or the malaria parasite, appeared to have predisposed the individuals to the high EBV loads observed (33–36). There are many differences, therefore, between such complex scenarios and clinically silent EBV acquisition in the nonimmunocompromised host, particularly that which occurs covertly in young adults in the developed world. Only one previous study, based in Australia, focused on asymptomatic primary infection in these circumstances (37). During prospective screening of volunteers for an early EBV vaccine trial, Silins et al. (37) serendipitously identified four healthy anti-VCA IgM-positive (IgM^+^), anti-VCA IgG-positive (IgG^+^) or IgG-negative (IgG^−^), and anti-EBNA1 IgG^−^ individuals, two of whom also had EBV genome loads in their blood like those seen in patients during the acute phase of IM. Although they were not analyzed for EBV-specific T cell or NK cell responses, all four asymptomatic seroconverters had peripheral blood mononuclear cell (PBMC) counts in the normal range, and only one showed by T cell receptor Vβ analysis evidence of clonal T cell expansions like those that occur in patients with IM (37). The present study set out to identify more such individuals, in this case, within a cohort of more than 400 medical students who volunteered blood samples at the beginning of their course of university study and, in some cases, annually over the following 4 years. All were monitored for serologic status with respect to two herpesviruses, EBV and cytomegalovirus (CMV), and for the EBV load in PBMCs. Overall, using a combination of serologic and virologic parameters, six subjects were identified to be likely undergoing asymptomatic primary infection with EBV at the time that a blood sample was taken; additional blood samples allowed prospective studies in five of these cases.

## RESULTS

### Identifying cases of asymptomatic primary infection.

**FIG 1:**
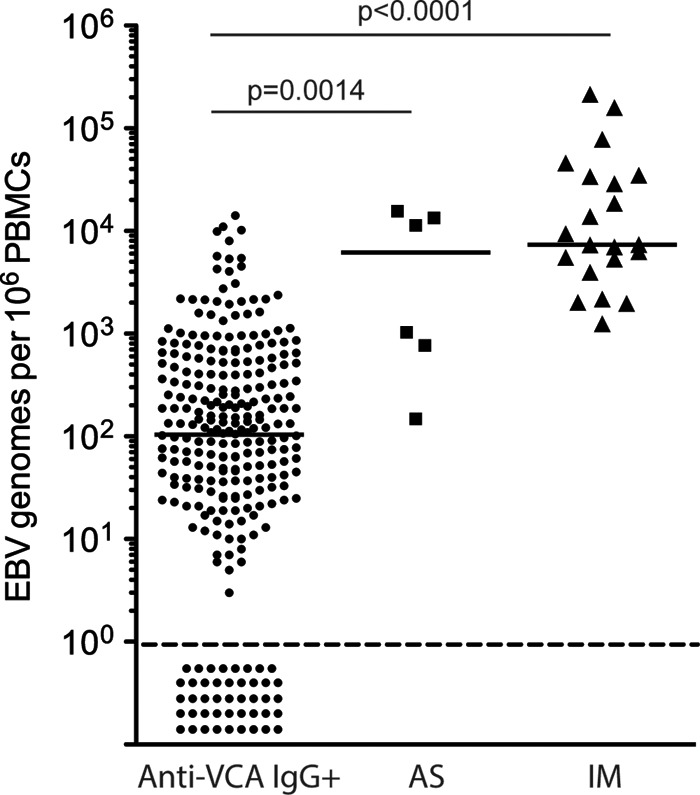
EBV genome loads in long-term virus carriers and cases of primary infection. Viral loads are reported as the number of EBV genome copies per 10^6^ PBMCs for 276 anti-VCA IgG^+^ virus carriers, six individuals with asymptomatic primary infection (AS1 to AS6) from the same student cohort, and 21 patients during the acute phase IM included for reference. Median viral loads (horizontal bars) for the 232 of 276 anti-VCA IgG^+^ virus carriers with detectable EBV DNA loads, for AS1 to AS6, and for the 21 IM patients were 103, 6,190, and 7,350 EBV genome copies per 10^6^ PBMCs, respectively. Data points below the dotted line identify the 44 anti-VCA IgG^+^ virus carriers who had undetectable EBV loads. Not shown are data from 166 anti-VCA IgG^−^ individuals in the same student cohort; all 166 had, as expected, undetectable viral loads.

A total of 448 medical school entrants, all in good health and with no recent history of infectious disease, volunteered to provide an initial blood sample. Of these, 278 (62%) were found to be anti-VCA IgG^+^, suggestive of an already established EBV carrier state. Viral DNA was detected in the majority of but not all seropositive cases (Fig. 1); this reflects the limitation of the assay when applied to DNA from 6 × 10^5^ PBMCs (36), since in our hands almost all seropositive individuals are detectably EBV genome positive in the same assay using DNA from 6 × 10^5^ purified peripheral blood B cells (10). As seen before among virus carriers in the UK population (10, 36), viral load values were spread across a very wide (10^4^-fold) range (Fig. 1). Indeed, that range slightly overlapped the range of viral loads seen in patients during the acute phase of IM, though the median value for patients during the acute phase of IM is about 70-fold higher than the median for those in the virus carrier state.

During the analysis of these initial blood samples or subsequently, during annual follow-ups, a combination of viral load assays and serologic screening identified six individuals (designated asymptomatic [AS] individuals AS1 to AS6) who had, fortuitously, been sampled while undergoing an asymptomatic primary infection. Four cases came to light through being EBV genome positive in the viral load assay yet serologically anti-VCA IgG^−^. Further antibody assays of the plasma samples taken at this time showed that two of these individuals (AS1 and AS5) were anti-VCA IgM^+^ and anti-EBNA1 IgG^−^ at the time, while the other two (AS3 and AS4) were still seronegative even for anti-VCA IgM. Interestingly, all four individuals had high to very high EBV genome loads in the blood (770 to 15,600 genomes per 10^6^ PBMCs) but remained asymptomatic in the weeks after the sample was taken. All four were studied prospectively either through the collection of additional voluntary blood samples or through the collection of samples biannually.

To search for other examples of occult primary infection, we retrospectively screened 248 plasma samples from the above-described anti-VCA IgG^+^ cohort and looked for cases with an anti-VCA IgM^+^, anti-EBNA1 IgG^−^ profile. Interestingly, 17 samples were anti-EBNA1 IgG^−^, but of these, 15 were also anti-VCA IgM negative (IgM^−^). We infer that these 15 anti-VCA IgM^−^, anti-VCA IgG^+^, anti-EBNA1 IgG^−^ cases (EBV load range, 26 to 4,536 genomes per 10^6^ PBMCs; median value, 312 genomes per 10^6^ PBMCs) reflected either recent but not current primary infections or the small fraction of virus carriers (estimated to be about 3%) who remain anti-EBNA1 IgG^−^ in the longer term (38). More importantly, we identified two individuals with an anti-VCA IgM^+^, anti-EBNA1 IgG^−^ profile indicative of primary infection, and both of these individuals remained well in the weeks following collection of the key blood sample. One individual, AS2, also had a very high viral load (11,350 genomes per 10^6^ PBMCs) and, because additional biannual samples were available, could be studied prospectively. The other individual, AS6, who had only a relatively low viral load (148 genomes per 10^6^ PBMCs), did not give additional samples and our observations are limited to just the one blood sample. Figure 1 compares the viral load data for all six AS cases at the time of asymptomatic infection with the values seen in long-term virus carriers (anti-VCA IgG^+^) and in patients during the acute phase of IM; the viral loads seen in individuals AS1 to AS6 were significantly higher than those seen in individuals in the carrier state and in three cases were well within the range for patients during the acute phase of IM.

For comparison, the same serologic assays were used to screen 21 patients during the acute phase of IM, whose EBV loads are also included in Fig. 1; all 21 were anti-VCA IgM^+^, and 14 were already anti-VCA IgG^+^, whereas all but 1 were anti-EBNA1 IgG^−^ (data not shown). Note, therefore, that the serological profiles in four of our asymptomatic cases (AS1, AS2, AS5, and AS6, who were anti-VCA IgM^+^, anti-VCA IgG^+^ or IgG^−^, and anti-EBNA1 IgG^−^) resemble those typically seen in patients during the acute phase of IM. In contrast, the profile seen in the two other cases (AS3 and AS4, who were anti-VCA IgM^−^, anti-VCA IgG^−^, and anti-EBNA1 IgG^−^) is atypical and implies that blood samples were collected these individuals at an earlier stage of infection, before the development of an IgM response. We also determined the CMV serostatus of the AS cohort and their acute IM comparators, given the possibility that preexisting CMV carriage might influence the course of primary EBV infection (39, 40). All six AS individuals were CMV seronegative at the time of EBV acquisition, and the five prospectively studied cases remained so throughout the whole study period. The majority (18/21) of IM cases were, likewise, CMV seronegative at the time of acute IM.

### Prospective studies of AS1 to AS5: viral load, antibody, and T cell responses.

**FIG 2:**
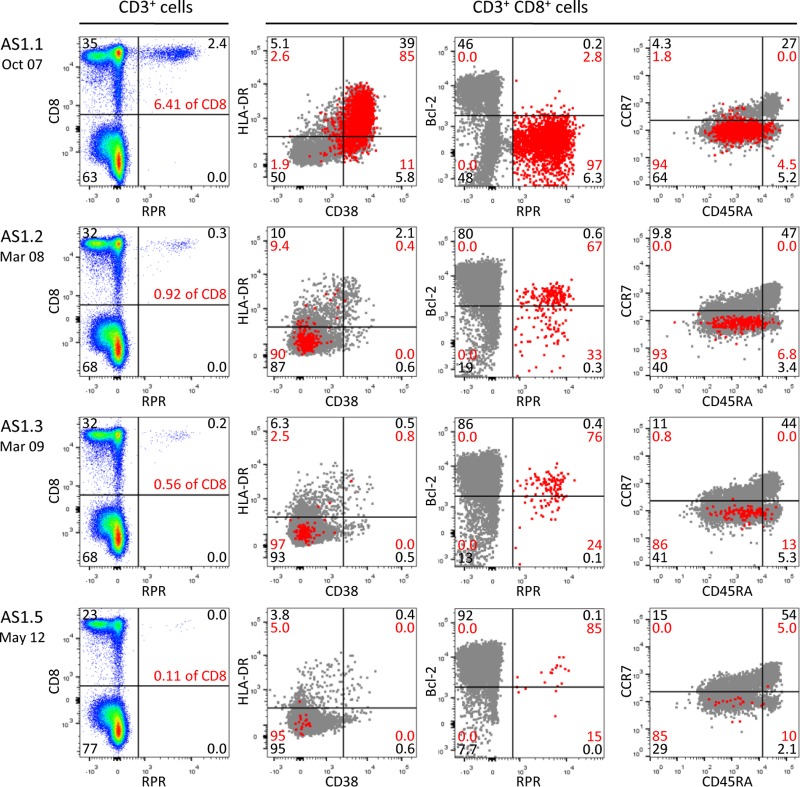
Longitudinal analysis of the B*0701-restricted RPR lytic epitope response in donor AS1. PBMCs were obtained from donor AS1 at the time of asymptomatic infection (AS1.1) and again 5, 17, and 55 months later (AS1.2, AS1.3, and AS1.5, respectively) and screened for responses against the B*0701-restricted RPR lytic epitope derived from BaRF1. The left-hand column shows the CD8/tetramer staining profiles of the CD3^+^ T cell population. The remaining plots show the phenotypic profiles of the whole CD8^+^ T cell population (gray dots) and of the RPR-specific CD8^+^ T cells (red dots) after costaining for HLA-DR and CD38, for Bcl2 and the RPR tetramer, and for CCR7 and CD45RA. Black and red numbers indicate the percent distribution of total CD3^+^ T cells and of RPR-specific cells across the four quadrants, respectively.

AS1 gave samples on four occasions, initially in October 2007 and then again 5, 17, and 55 months after that date. This individual was identified to have a very high viral load (15,600 genomes per 10^6^ PBMCs) at the time of collection of the first blood sample and a serological picture associated with primary infection, i.e., anti-VCA IgM^+^, anti-VCA IgG^−^, and anti-EBNA1 IgG^−^ (Table 1). Surprisingly, the viral load in blood was still high at 5 months, by which time both anti-VCA IgG and anti-EBNA1 IgG antibody responses had developed, whereas by later times, the viral load in blood had fallen to a stable level (albeit at the high end of the virus carrier range) and the serologic picture was that of a long-term virus carrier, i.e., anti-VCA IgM^−^, anti-VCA IgG^+^, and anti-EBNA1 IgG^+^. In contrast to the situation typically seen in patients during the acute phase of IM, AS1's lymphocyte count was not raised at the time of primary infection, and there was no dramatic expansion of CD8^+^ T cells. However, the 30% CD8^+^ T cell representation among the lymphocyte population was higher than that seen in blood samples collected from this individual at later times. Furthermore, almost 40% of the circulating CD8^+^ T cells in the blood sample collected at the time of primary infection had an activated CD38^+^ HLA-DR^+^ phenotype, suggesting that a mild expansion had occurred. Since AS1 was HLA-B*0702 positive, this allowed the initial and subsequent blood samples to be examined for EBV-specific CD8^+^ T cells using tetramers for three EBV-coded B*0702 -restricted epitopes, two lytic (RPR, TPS) and one latent (RPP), here listed in their typically observed order of dominance (RPR > RPP > TPS) (epitopes are designated by their initial three residues; see Materials and Methods for the complete sequences of the epitopes). More than 6% of all CD8^+^ T cells had an RPR-specific response at the time of primary infection (Fig. 2; Table 1), and just as seen for EBV-specific effectors during the acute phase of IM (15, 16), the great majority of these RPR-specific cells were in the CD45RA^−^ CCR7^−^ subset and had an activated CD38^+^ HLA-DR^+^ phenotype with the associated downregulation of Bcl2 (Fig. 2). Overall, therefore, about 14% of the CD8^+^ T cell activation that occurred during primary infection of AS1 could be explained by a response to this one B*0702-restricted epitope. Five months later, the general CD8^+^ T cell activation had largely resolved and the RPR epitope response had contracted to a lower level with a loss of activation markers (Fig. 2; Table 1). By that time, responses to the subdominant RPP and TPS epitopes had developed, with the latter still having remnants of an activated phenotype (Table 1). Subsequent blood samples showed a further slow fall in the size of all three B*0702-restricted epitope responses and a complete loss of activation markers.

**TABLE 1:** Longitudinal viral loads, CD8 responses, and serological profiles of donors AS1 and AS2

Donor^*a*^	Time (mo)^*b*^	EBV load^*c*^	Lymphocyte count (10^6^/ml)	% CD8^+^ cells^*d*^	% activated CD8^+^ cells^*e*^	EBV CD8 responses			EBV serology		
						EBV tetramer	% tetramer-positive CD8^+^ cells	% activated tetramer-positive CD8^+^ cells	EBV VCA IgM titer	EBV VCA IgG titer	EBNA1 IgG index value
AS1	0	15,600	1.3	30	39.0	RPR	6.41	85.0	1/20	Negative	Negative
						RPP	0	0			
						TPS	0	0			
	+5	6,020	1.7	25	2.1	RPR	0.92	0.4	1/20	1/320	3.9
						RPP	0.47	5.9			
						TPS	0.20	21.6			
	+17	847	3.4	22	0.5	RPR	0.56	0.8	Negative	1/320	16.3
						RPP	0.34	0			
						TPS	0.14	9.7			
	+55	140	1.6	18	0.4	RPR	0.11	0	Negative	1/320	12.8
						RPP	0.08	0			
						TPS	0.06	0			
AS2	−27	0	2.3	27	1.6	YVL	0	0	Negative	Negative	Negative
						GLC	0	0			
						LLI	0	0			
						CLG	0	0			
	0	11,350	4.1	46	45.0	YVL	1.13	96.0	1/5	1/80	Negative
						GLC	0.38	94.0			
						LLI	0.09	72.0			
						CLG	0	0			
	+25	350	2.8	27	0.5	YVL	0.07	3.1	Negative	1/320	14.5
						GLC	0.09	3.9			
						LLI	0.02	0			
						CLG	0	0			

a Donor HLA types: for AS1, A1, A3, B7, and B13; for AS2, A2, A11, B40, and B44.

b Time (in months) since primary infection.

c Number of EBV genome copies per 10^6^ PBMCs.

d Percentage of CD8^+^ cells among total lymphocytes.

e Percentage of HLA-DR^+^ CD38^+^ CD8^+^ cells among total CD8^+^ cells.

**FIG 3:**
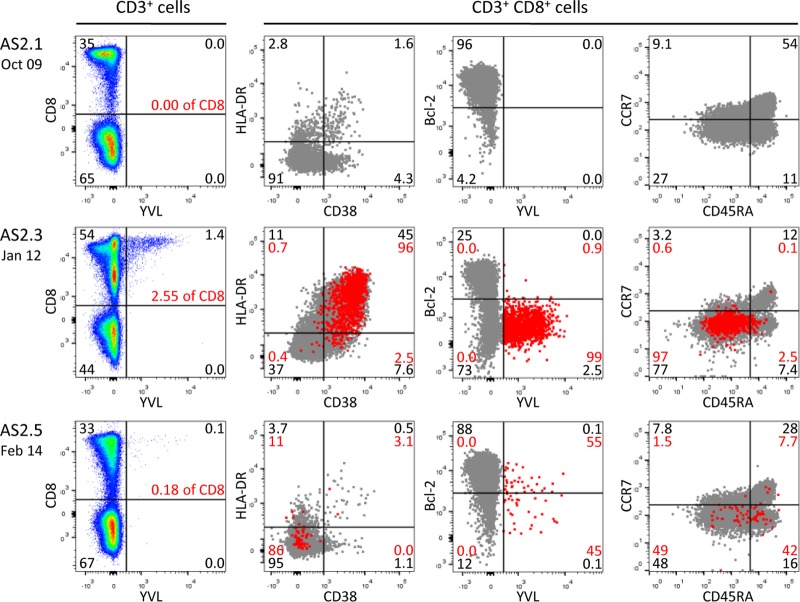
Longitudinal analysis of the A*0201-restricted YVL lytic epitope response in donor AS2. PBMCs were obtained from donor AS2 27 months before infection (AS2.1), at the time of asymptomatic infection (AS2.3), and again 25 months later (AS2.5) and screened for responses against the A*0201-restricted YVL lytic epitope from BRLF1. The left-hand column shows the CD8 tetramer staining profiles of the CD3^+^ T cell population. The remaining plots show the phenotypic profiles of the whole CD8^+^ T cell population (gray dots) and of the YVL-specific CD8^+^ T cells (red dots) after costaining for HLA-DR and CD38, for Bcl2 and the YVL tetramer, and for CCR7 and CD45RA. Black and red numbers indicate the percent distribution of total CD3^+^ T cells and of YVL-specific cells across the four quadrants, respectively.

AS2 was EBV negative by both viral load and serology at the time that the first blood sample was collected in October 2009 but 2 years later was found to have a very high viral load (11,350 genomes per 10^6^ PBMCs) and an anti-VCA IgM^+^, anti-VCA IgG^+^, anti-EBNA1 IgG^−^ antibody profile typical of that seen during a primary infection (Table 1). When AS2 was sampled a further 2 years on, the viral load had fallen to a value just above the median for normal carriers and the serological picture, anti-VCA IgM^−^, anti-VCA IgG^+^, anti-EBNA1 IgG^+^, was typical of that for individuals in the long-term carrier state. In this case, the lymphocyte count at the time of primary infection was slightly raised (by a factor of 1.5- to 1.8-fold) over earlier and later values. Furthermore, the percentage of CD8^+^ T cell representation in the lymphocyte population was increased almost 2-fold, 45% of those CD8^+^ T cells were activated, and at least some of those T cells were EBV specific (Table 1). Thus, tetramer staining identified a highly activated (CD38^+^ HLA-DR^+^ Bcl2^low^) CD8^+^ T cell response to the HLA-A*0201-restricted EBV lytic cycle epitope YVL (Fig. 3). These YVL-specific cells made up 2.5% of all CD8^+^ T cells in the blood at the time and >5% of the activated population. Two years later, YVL-specific cells were detectable in lower numbers and had lost their activation markers. During primary infection, the YVL response was also accompanied by smaller, but similarly activated, responses to two other A*0201-restricted lytic epitopes, GLC and LLI (Table 1); these again entered memory as smaller populations. No detectable response to CLG, a weak A*0201-restricted latent cycle epitope, ever developed.

Corresponding findings for two further individuals, AS3 and AS4, are summarized in Table 2. These were distinct from the cases described above, in that both were identified to have viral loads that were moderately high (770 and 1,030 genomes per 10^6^ PBMCs, respectively) yet at a time when both were still anti-VCA IgM^−^, anti-VCA IgG^−^, anti-EBNA1 IgG^−^. This implies that these two individuals had been caught slightly earlier in the course of infection (relative to AS1 and AS2), although, clearly, EBV had already entered the circulating B cell pool. At this time, AS3 (HLA-A*0201 and B*0702 positive) showed no evidence of raised lymphocyte counts, of any expansion or activation in the CD8^+^ T cell pool, or of any A*0201 or B*0702 EBV epitope reactivity (Table 2). A T cell response did subsequently occur, however, since in blood samples taken 2 and 4 years later, tetramer staining detected small nonactivated memory populations specific for several A*0201- and B*0702-restricted epitopes; the same blood samples taken at later times also confirmed that the EBV load had fallen, eventually to below detectable levels, and conversion to a typical virus carrier serostatus (anti-VCA IgM^−^, anti-VCA IgG^+^, anti-EBNA1 IgG^+^). In the case of AS4 (HLA-B*0702 positive), total lymphocyte and CD8^+^ T cell numbers were likewise unchanged at the time of primary infection relative to those seen in earlier and later blood samples, and there was minimal activation of the general CD8^+^ T cell pool. Interestingly, however, tetramer staining did detect a small population of cells specific for the B*0702-restricted RPR epitope, and 35% of these were activated. Responses to the subdominant B*0702-restricted epitopes RPP and TYS were not yet present, but 2 years later, all three reactivities were detectable in memory; by this time, the viral load in PBMCs had fallen below detectable levels and the serologic picture was typical of that of a long-term virus carrier (Table 2).

**TABLE 2:** Longitudinal viral loads, CD8 responses, and serological profiles of donors AS3 and AS4

Donor^*a*^	Time (mo)^*b*^	EBV load^*c*^	Lymphocyte count (10^6^/ml)	% CD8^+^ cells^*d*^	% activated CD8^+^ cells^*e*^	EBV CD8 responses			EBV serology		
						EBV tetramer	% tetramer-positive CD8^+^ cells	% activated tetramer-positive CD8^+^ cells	EBV VCA IgM titer	EBV VCA IgG titer	EBNA1 IgG index value
AS3	0	770	1.9	18	0.5	RPR	0	0	Negative	Negative	Negative
						TPS	0	0			
						RPP	0	0			
						YVL	0	0			
						GLC	0	0			
						LLI	0	0			
						CLG	0	0			
	+27	420	1.9	21	1.0	RPR	0.26	0	Negative	1/40	2.8
						TPS	0.05	0			
						RPP	0.05	0			
						YVL	0.06	0			
						GLC	0.06	7.7			
						LLI	0	0			
						CLG	0	0			
	+51	0	1.8	24	0.2	RPR	0.12	0	Negative	1/40	5.1
						TPS	0.01	0			
						RPP	0.02	0			
						YVL	0.03	0			
						GLC	0.06	0			
						LLI	0	0			
						CLG	0	0			
AS4	−9	0	1.9	24	0.4	RPR	0.00	0.0	Negative	Negative	Negative
						TPS	0.00	0.0			
						RPP	0.00	0.0			
	0	1,030	1.8	25	1.9	RPR	0.33	35.3	Negative	Negative	Negative
						TPS	0.00	0.0			
						RPP	0.00	0.0			
	+26	0	2.0	26	1.2	RPR	0.07	0.0	Negative	1/80	30.1
						TPS	0.01	0.0			
						RPP	0.50	1.5			

a Donor HLA types: for AS3, A1, A2, B7, and B57; for AS4, A24, A25, B7, and B18.

b Time (in months) since primary infection.

c Number of EBV genome copies per 10^6^ PBMCs.

d Percentage of CD8^+^ cells among total lymphocytes.

e Percentage of HLA-DR^+^ CD38^+^ CD8^+^ cells among total CD8^+^ cells.

**FIG 4:**
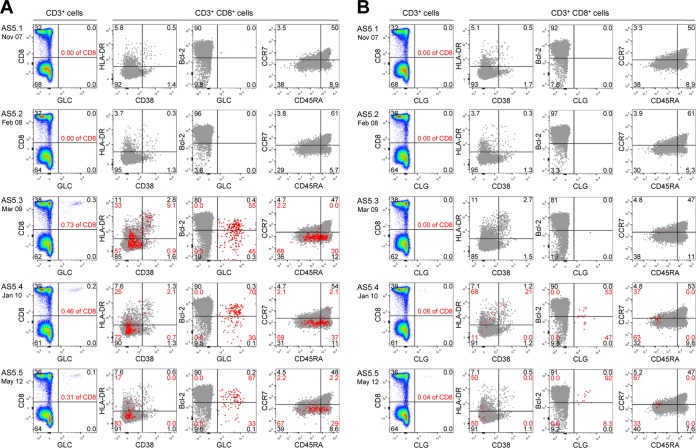
Longitudinal analysis of the delayed EBV lytic epitope responses in donor AS5. PBMCs were obtained from donor AS5 at the time of asymptomatic infection (AS5.1) and again 3, 16, 26, and 54 months later (AS5.2, AS5.3, AS5.4, and AS5.5, respectively) and screened for responses against the A*0201-restricted GLC lytic epitope from BMLF1 (A) and the A*0201-restricted CLG latent epitope from LMP2 (B). In each case, the left-hand column shows the CD8 tetramer staining profiles of the CD3^+^ T cell population. The remaining plots show the phenotypic profiles of the whole CD8^+^ T cell population (gray dots) and of the tetramer-specific CD8^+^ T cells after costaining for HLA-DR and CD38, for Bcl2 and the relevant tetramer, and for CCR7 and CD45RA. Black and red numbers indicate the percent distribution of total CD3^+^ T cells and of GLC- or CLG-specific cells across the four quadrants, respectively.

Donor AS5 was sampled on 5 occasions, initially in November 2007 and then again 3, 16, 26, and 54 months after that date. At the time of collection of the first blood sample, this individual (like AS1) was identified to have a very high viral load (13,450 genomes per 10^6^ PBMCs) and a serologic picture (anti-VCA IgM^+^, anti-VCA IgG^−^, anti-EBNA1 IgG^−^) associated with primary infection (Table 3). In this case, the viral load in the blood had fallen well into the normal range for carriers within 3 months, remained at that level at 16 months, and then became undetectable at later blood collection times. However, the serologic profile was slow to change; AS5 still remained anti-VCA IgM^+^, anti-VCA IgG^−^, anti-EBNA1 IgG^−^ at 3 months, had become anti-VCA IgM^−^, anti-VCA IgG^+^, anti-EBNA1 IgG^−^ at 16 months, and did not acquire the typical anti-VCA IgM^−^, anti-VCA IgG^+^, anti-EBNA1 IgG^+^ carrier profile until 26 months. Interestingly, the T cell response was also slow to develop (Table 3). There was no sign of the expansion or activation of the circulating CD8^+^ T cell population either at the time of primary infection or 3 months later. Total lymphocyte counts were similar at these times but then increased to a higher level that was maintained at the last three blood collection times, though without any change in the percentage of CD8^+^ T cells. As AS5 was HLA-A*0201 positive, we stained successive samples using A*0201 tetramers with relevant EBV epitopes. Responses to the lytic epitopes GLC (Fig. 4), YVL, and LLI were absent in the first two blood samples collected but were first detected at 16 months postinfection as small populations still bearing some residual evidence of an activated phenotype (Table 3). Their numbers had fallen at 26 months, by which time a partly activated response to the subdominant latent epitope CLG (Fig. 4) was also in evidence, and the responses to all four epitopes had assumed typical long-term memory levels by the time of collection of the last blood sample.

**TABLE 3:** Longitudinal viral loads, CD8 responses, and serological profiles of donor AS5^*a*^

Time (mo)^*b*^	EBV load^*c*^	Lymphocyte count (10^6^/ml)	% CD8^+^ cells^*d*^	% activated CD8^+^ cells^*e*^	EBV CD8 responses			EBV serology		
					EBV tetramer	% tetramer-positive CD8^+^ cells	% activated tetramer-positive CD8^+^ cells	EBV VCA IgM titer	EBV VCA IgG titer	EBNA1 IgG index value
0	13,450	1.4	26	0.5	YVL	0	0	1/20	Negative	Negative
					GLC	0	0			
					LLI	0	0			
					CLG	0	0			
+3	180	1.4	31	0.3	YVL	0	0	1/10	Negative	Negative
					GLC	0	0			
					LLI	0	0			
					CLG	0	0			
+16	160	3.9	32	2.8	YVL	0.69	6.9	Negative	1/160	Negative
					GLC	0.73	9.1			
					LLI	0.73	4.3			
					CLG	0	0			
+26	0	3.3	32	1.3	YVL	0.26	5.0	Negative	1/160	2.9
					GLC	0.46	2.1			
					LLI	0.20	1.5			
					CLG	0.06	21.0			
+54	0	3.4	30	0.6	YVL	0.23	0	Negative	1/160	1.7
					GLC	0.31	0			
					LLI	0.16	0			
					CLG	0.04	0			

a Donor HLA type: A2, A31, B40, and B44.

b Time (in months) since primary infection.

c Number of EBV genome copies per 10^6^ PBMCs.

d Percentage of CD8^+^ cells among total lymphocytes.

e Percentage of HLA-DR^+^ CD38^+^ CD8^+^ cells among total CD8^+^ cells.

To allow comparison of these individuals with asymptomatic EBV infections and individuals with classical IM, we selected four IM patients (IM221, IM232, IM253, and IM265) who were either HLA-A*0201 or HLA-B*0702 positive and from whom samples obtained during the acute phase and follow-up were available for retrospective analysis. These samples were assayed alongside the samples from individuals with symptomatic infection using the same reagents, and the results are summarized in Table 4. At the time of the acute phase of IM, all four patients had high viral loads (1,270 to 21,650 genome copies/ml), while the three from whom acute-phase serum samples were available had an antibody profile consistent with that seen during primary infection (anti-VCA IgM^+^, anti-VCA IgG^+^, anti-EBNA1 IgG^−^). All four had elevated lymphocyte counts (3.3 × 10^6^ to 8.8 × 10^6^ cells/ml) with a high percentage (60 to 76%) of CD8^+^ T cells and with 77 to 95% of those CD8^+^ T cells having an activated phenotype. In blood samples collected later, the viral load fell (in three of four cases), the antibody profile had become anti-VCA IgM^−^, anti-VCA IgG^+^, anti-EBNA1 IgG^+^, and the lymphocyte count normalized, as did the percentage of CD8^+^ T cells and the CD8 activation phenotype. Staining with the above-described panel of A*0201 and B*0702 tetramers showed activated CD8 responses to the relevant epitopes during the acute phase, followed by the resolution of these activated responses down to resting memory levels (Table 4). Note that in the acute phase these particular epitope-specific responses were relatively small (comprising only up to 7% of total CD8^+^ T cells), reflecting the fact that neither HLA-A*0201 nor B*0702 is a strong EBV response allele and is frequently outcompeted by more dominant restricting alleles in the genotype, most likely by HLA-B*0801 in the cases of IM221 and IM232 (15, 16).

**TABLE 4:** Longitudinal viral loads, CD8 responses, and serological profiles of acute IM cases

Donor^*a*^	Time (mo)^*b*^	EBV load^*c*^	Lymphocyte count (10^6^/ml)	% CD8^+^ cells^*d*^	% activated CD8^+^ cells^*e*^	EBV CD8 responses			EBV serology^*f*^		
						EBV tetramer	% tetramer-positive CD8^+^ cells	% activated tetramer-positive CD8^+^ cells	EBV IgM VCA titer	EBV IgG VCA titer	IgG EBNA1 index value
IM221	0	1,270	3.3	76	83.1	YVL	2.10	97.4	1/40	1/80	Negative
						GLC	0.07	57.6			
						LLI	1.92	90.9			
						CLG	0	0			
	+19	640	1.1	35	10.8	YVL	0.46	16.1	Negative	1/160	19.4
						GLC	0.20	7.1			
						LLI	0.46	11.0			
						CLG	0.13	17.4			
	+29	130	0.7	32	8.2	YVL	0.54	14.5	Negative	1/80	16.8
						GLC	0.26	17.4			
						LLI	0.29	17.2			
						CLG	0.06	10.5			
IM232	0	19,290	8.8	74	80.3	YVL	0.05	87.2	Not done		
						GLC	0.34	96.4			
						LLI	0	0			
						CLG	0	0			
	+15	49	1.8	29	0.9	YVL	0.06	0	Negative	1/160	1.7
						GLC	1.80	1.8			
						LLI	0.02	0			
						CLG	0.03	0			
	+24	111	1.5	25	0.6	YVL	0.05	7.1	Negative	1/640	2.1
						GLC	0.75	1.5			
						LLI	0	0			
						CLG	0	0			
	+76	0	2.5	28	0.4	YVL	0.07	0	Negative	1/640	3.5
						GLC	1.36	0.3			
						LLI	0.01	0			
						CLG	0.03	0			
IM253	0	5,010	5.0	76	95.3	RPR	7.07	98.7	1/320	1/160	Negative
						TPS	0	0			
						RPP	0.44	99.5			
	+7	8,740	0.9	37	6.1	RPR	1.77	2.3	Negative	1/160	16.0
						TPS	0.74	26.3			
						RPP	2.23	4.1			
	+19	5,800	0.7	28	6.9	RPR	1.03	2.7	Negative	1/80	31.5
						TPS	0.55	8.2			
						RPP	1.39	4.1			
IM265	0	21,650	3.3	60	76.9	RPR	3.62	91.5	1/40	1/80	Negative
						TPS	0	0			
						RPP	1.21	99.5			
	+5	5,420	0.6	26	2.2	RPR	0.70	2.7	Negative	1/80	4.8
						TPS	0.25	18.5			
						RPP	0.30	17.7			
	+19	1,310	1.2	27	1.4	RPR	0.23	4.8	Negative	1/160	21.0
						TPS	0.24	3.1			
						RPP	0.29	7.7			

a Donor HLA types: for IM221, A1, A2, B8, and B44; for IM232, A2, A3, B8, and B27; for IM253, A3, A24, B7, and B37; for IM265, A23, A26, B7, and B44.

b Time (in months) since primary infection.

c Number of EBV genome copies per 10^6^ PBMCs.

d Percentage of CD8^+^ cells among total lymphocytes.

e Percentage of HLA-DR^+^ CD38^+^ CD8^+^ cells among total CD8^+^ cells.

f IM221 and IM253 were CMV seronegative, IM265 was CMV seropositive, and IM232 was not tested for CMV.

**FIG 5:**
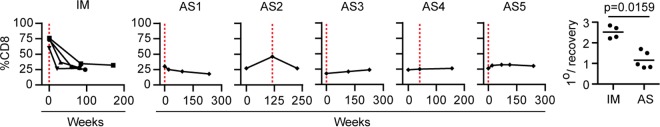
Longitudinal analysis of CD8^+^ T cells in IM patients and in cases of asymptomatic infection. Shown are the proportions of CD8^+^ T cells within the lymphocyte population over time for four IM patients (IM221, IM232, IM253, IM265) (left) and for asymptomatic infection cases AS1 to AS5 (the five middle graphs). In each case, the time of primary infection is indicated by the vertical red dotted line. The scatter plots (right) show the ratio of the proportion of CD8^+^ T cells at the time of primary infection to the mean proportion of CD8^+^ T cells at time points more than 6 months later (1°/recovery) for both the IM and AS groups.

The data in Fig. 5 show how the percentage of CD8^+^ T cells within the lymphocyte population changed over time for these IM patients and for asymptomatic cases AS1 to AS5; red dotted lines denote the time of primary infection. The scatter plots (Fig. 5, right) show for each of the IM patients and for AS1 to AS5 the ratio of the percentage of CD8^+^ T cells seen in primary infection versus the mean percentage seen in blood samples taken from the same individual >6 months postinfection. The significant difference between the two sets of results makes it clear that the gross expansion of CD8^+^ T cells typically seen in patients during the acute phase of IM was not observed during asymptomatic infection. Note that a similar picture emerged from the other case of asymptomatic primary infection, AS6, from whom only the primary infection blood sample was available. There was again no obvious lymphocytosis in the blood, and the CD8^+^ T cell population constituted only 25% lymphocytes and completely lacked activation markers (data not shown).

### Prospective studies of AS1 to AS5: NK cell responses.

**FIG 6:**
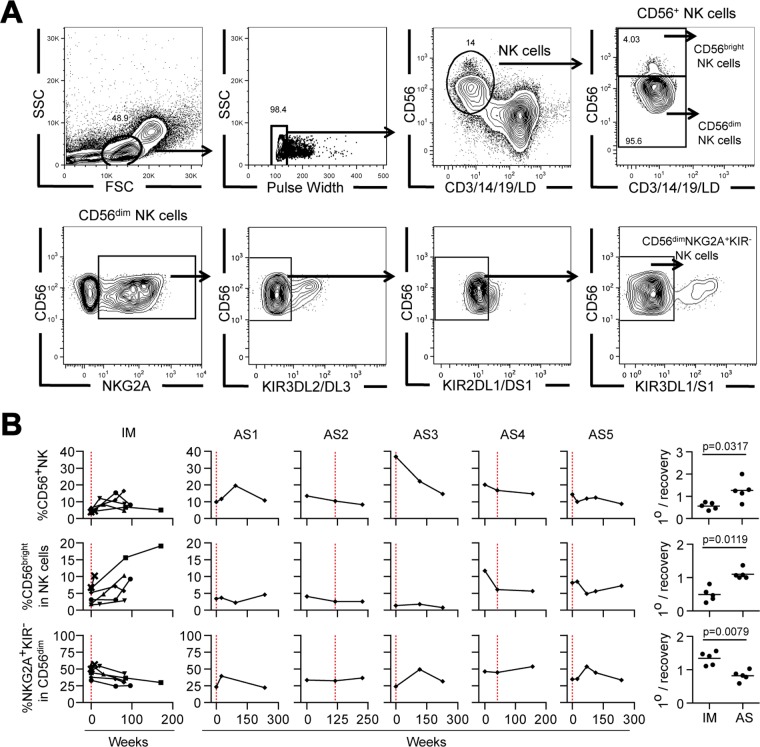
Longitudinal analysis of NK cell subsets in IM patients and in cases of asymptomatic infection. (A) Gating strategies for flow cytometric analysis to identify total NK cells (defined as live lineage-negative CD56^+^ cells), CD56^bright^ cells, CD56^dim^ cells, and CD56^dim^ NKG2A^+^ KIR^−^ cells within the lymphocyte population. The percentage of cells in each gate is indicated. The staining shown is for a sample from a representative asymptomatic subject (AS2) collected 27 months before the detection of viremia. FSC, forward scatter; SSC, side scatter. (B) Longitudinal blood samples from five IM patients (IM221, IM225, IM232, IM253, and IM265) and asymptomatic infection cases AS1 to AS5 were analyzed by multiparameter flow cytometry to determine the percentage of CD56^+^ NK cells within lymphocytes, the percentage of CD56^bright^ NK cells within the circulating CD56^+^ NK population, and the percentage of NKG2A^+^ KIR^−^ cells in the CD56^dim^ NK cell population. Combined results for IM patients and individual results for AS1 to AS5 are displayed as shown in Fig. 5, with the time of primary infection being identified by a vertical red dotted line. The scatter plots (right-hand column) show the ratio of the proportion of each subset at the time of primary infection to the mean proportion at time points more than 6 months later (1°/recovery) for both the IM and AS groups.

Given recent reports of mild NK cell expansion (19) accompanied by differentiation of immature CD56^bright^ cells to an intermediate CD56^dim^ NKG2A^+^ KIR^−^ phenotype (19–21) in patients during the acute phase of IM, we established staining protocols that could identify the total NK cell population and the relevant NK cell subsets (Fig. 6A). We then used these protocols to analyze the sequential samples from patients during the acute phase of IM and AS donors. Figure 6B shows the individual data and summary plots in a format that follows the layout introduced in Fig. 5.

Studying the PBMCs collected from five prospectively studied IM patients (including the four used for tetramer staining as described above) during and after the acute phase confirmed the recently reported trends (19–21). Note that the size of the CD56^+^ NK cell population, when expressed as a percentage of total lymphocytes, was typically about 2-fold lower in blood samples collected during the acute phase of IM than in blood samples collected later, after the acute phase of IM; however, this reduced percentage during the acute phase is a side effect of the dominant CD8^+^ T cell expansion and does not reflect a real fall in numbers. Indeed, when allowance is made for the 3- to 4-fold increase in total PBMCs seen during IM, absolute NK cell numbers were 1.5- to 2-fold higher during the acute phase. Staining with the NK cell subset markers further showed that the proportion of CD56^bright^ cells within the NK cell population was lower during the acute phase than after the acute phase of IM, while that of CD56^dim^ NKG2A^+^ KIR^−^ cells was higher (Fig. 6B), consistent with a preferential expansion of the latter subset during IM (19–21).

In contrast, corresponding data on sequential blood samples from donors AS1 to AS5 showed, in most cases, no evidence of elevated CD56^+^ NK cell representation within the lymphocyte population during primary infection and no change in the relative representation of the CD56^bright^ and CD56^dim^ NKG2A^+^ KIR^−^ subsets (Fig. 6B). The one partial exception was AS3, in whom the percentage of CD56^+^ cells within the (nonexpanded) PBMC pool at the time of primary infection was unusually high, whereas more typical values were seen in later blood samples. However, this apparent expansion occurred without enrichment of the CD56^dim^ NKG2A^+^ KIR^−^ NK subset (Fig. 6B). Furthermore, in their blood samples collected at the time of primary infection, the AS donors showed no evidence of NK cell activation using two markers, increased HLA-DR^+^ expression and the loss of Bcl2, that, in our hands, were typically seen in the NK cells of patients during the acute phase of IM (data not shown). The summary scatter plots (Fig. 6B, center right and bottom right) show the percentage of CD56^bright^ and CD56^dim^ NKG2A^+^ KIR^−^ NK cell subsets at the time of primary infection relative to that seen in later blood samples from the same individuals. The scatter plots for patients during the acute phase of IM and the AS individuals were significantly different, reflecting the absence of a detectable NK cell response like that seen in patients during the acute phase of IM at the time that samples were collected from the five asymptomatic individuals with primary infections.

### Prospective studies of AS1 to AS5: DC frequencies and plasma cytokines.

The final sets of experiments asked to what extent two other hematological features of the acute phase of IM were detectable in individuals with asymptomatic primary infection, namely, the almost complete loss of pDCs and the partial loss of mDCs recently reported to occur in the blood of patients with IM (20, 22) and the well-documented increase in the circulating levels of various antiviral and/or proinflammatory cytokines and chemokines during the acute phase of disease (23–26).

**FIG 7:**
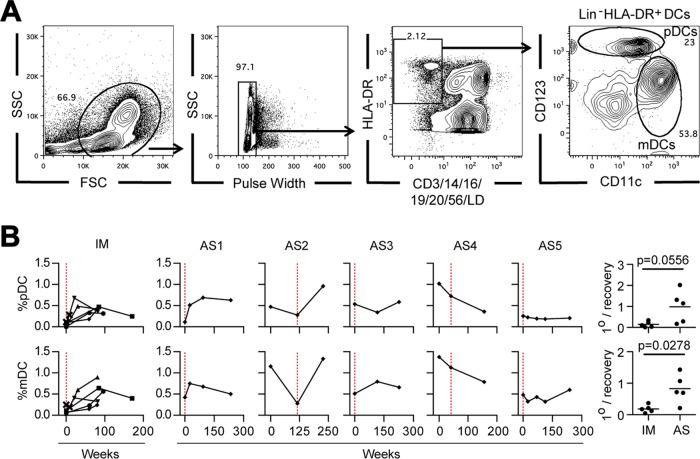
Longitudinal analysis of DC subsets in IM patients and in cases of asymptomatic infection. (A) Gating strategy for flow cytometric analysis of DC populations to identify total mDCs (defined as live lineage-negative [Lin^−^] HLA-DR^+^ CD11c^high^ CD123^−^ cells) and pDCs (defined as live lineage-negative HLA-DR^+^ CD11c^low^ CD123^high^ cells) withing the mononuclear cell population. The percentage of cells in each gate is indicated. The staining shown is for a sample from a representative asymptomatic subject (AS2) collected 27 months before the detection of viremia. (B) Longitudinal blood samples from five IM patients (IM221, IM225, IM232, IM253, and IM265) and asymptomatic infection cases AS1 to AS5 were analyzed by multiparameter flow cytometry to determine the percentage of pDCs and mDCs among circulating mononuclear cells. Combined results for IM patients and individual results for AS1 to AS5 are displayed as shown in Fig. 5, with the time of primary infection being identified by a vertical red dotted line. The scatter plots (right) show the ratio of the proportion of each subset at the time of primary infection to the mean proportion observed at time points more than 6 months later (1°/recovery) for both the IM and AS groups.

To examine DC populations, we established a staining protocol that identifies both pDCs (defined as live lineage-negative HLA-DR^+^ CD11c^low^ CD123^high^ cells) and mDCs (defined as live lineage-negative HLA-DR^+^ CD11c^high^ CD123^−^ cells) (Fig. 7A). Applying this to prospective blood samples from our five reference IM patients, we found that the percentage of pDCs and mDCs was very low (Fig. 7B, left). This was particularly the case for pDCs, with a median value of 0.062% mononuclear cells, 8-fold lower than the levels reached after the acute phase of IM, while mDCs underwent a similar but less marked decrease, with a median value of 0.065%, 4.5-fold lower than the levels observed after the acute phase of IM. A change of this magnitude cannot be wholly explained by the diluting effect of CD8^+^ T cell expansion in the blood of patients during the acute phase of IM but must reflect an actual depletion of the circulating pDC and mDC population. Corresponding data for AS1 to AS5 revealed a mixed picture (Fig. 7B). Both AS1 and AS2, the two individuals with asymptomatic infections characterized by high viral loads and an activated CD8^+^ T cell response, gave results suggestive of pDC and mDC depletion. In contrast, the other three asymptomatic infections occurred without obvious effects on circulating DC numbers. The summary scatter plots (Fig. 7B, right) illustrate the rather tight patterns of DC depletion seen in patients during the acute phase of IM relative to the values after the acute phase of IM versus the much more heterogeneous picture seen in the AS subjects.

**FIG 8:**
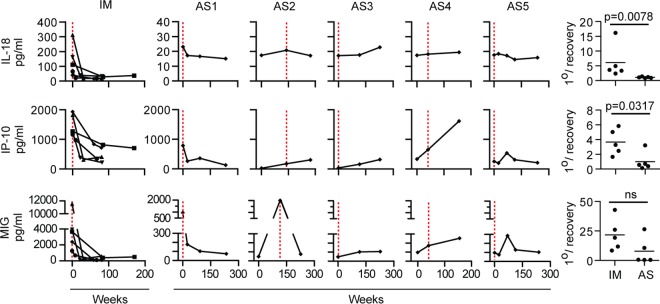
Longitudinal analysis of IL-18, IP-10, and MIG in plasma samples from IM patients and in individuals with asymptomatic infection. Longitudinal PBMC samples from five IM patients (IM221, IM225, IM248, IM253, and IM265) and asymptomatic infection cases AS1 to AS5 were analyzed for the concentrations of IL-18, IP-10, and MIG in plasma. Combined results for IM patients and individual results for AS1 to AS5 are arranged as shown in Fig. 5, with the time of primary infection being identified by a red dotted line. The scatter plots (right) show the ratio of the quantity of each analyte at the time of primary infection to the mean quantity observed at time points more than 6 months later (1°/recovery) for both the IM and AS groups. ns, not significant.

Finally, using a Luminex assay, we screened longitudinal plasma samples from the IM and AS cases for concentrations of 11 cytokines/chemokines reported to be elevated in patients IM. We found increased levels of IL-18, IP-10, and MIG to be the most sensitive and consistent indicators of analyte dysregulation in patients during the acute phase of IM, with peak concentrations being the greatest for MIG and IP-10. Figure 8 shows data from five IM patients, with the IL-18, MIG, and IP-10 levels present during the acute phase being much higher than those present in the weeks and months after the acute phase of IM. The corresponding results for the individual AS subjects are shown alongside; the picture was, again, heterogeneous. Both AS1 and AS2 had clear increases in MIG levels at the time of primary infection, accompanied by a small elevation of the IL-18 levels above the baseline levels (note the reduced scale for IL-18 and MIG concentrations compared to those seen in IM patients); in addition, AS1 showed an increase in IP-10 levels. In contrast, AS3 and AS4 showed rising levels of all 3 cytokines over time but no clear evidence of changes specifically linked to EBV infection. Most interesting were the data from AS5, for whom there was a long delay between virus acquisition and a virus-specific T cell response; in this case, increased levels of MIG and IP-10 coincident with the cellular response and not with infection *per se* were observed.

## DISCUSSION

Our understanding of primary EBV infection in its usual asymptomatic form is very limited because of the difficulty of studying a clinically silent event. As a result, much is inferred from the study of IM, a disease which appeared in affluent societies as an unexpected consequence of delayed primary infection and which, in evolutionary terms, is a novelty. Whether IM is a legitimate model for the successful control of primary EBV infection or an artifact of inefficient control remains to be determined. The fact that not all delayed primary infections are clinically manifest as IM (3–6) affords an opportunity to address this issue, provided that one can identify asymptomatic cases. An earlier study (37) serendipitously identified four IgM anti-VCA-positive individuals during serologic screening of volunteers for an EBV vaccine trial. In the present work, we took advantage of a study in which entrants into a UK medical school were screened annually or biannually throughout their degree course for EBV and CMV status. This allowed us to identify six individuals undergoing subclinical primary infection with EBV at one point during their studies; four of these were identified to be anti-VCA IgM^+^ by serologic screening (as in reference 37), while two were found to be EBV DNA positive yet still anti-VCA IgM negative (IgM^−^). In two cases (both anti-VCA IgM^+^), we were able to arrange for the collection of additional blood samples in the months following infection; in three other cases, we had access only to those samples taken biannually before or after the event, and in one case, we had only the blood sample collected at the time of primary infection. Such constraints are clearly a limitation since changes are occurring rapidly during primary infection and one's snapshot of the virus-host interaction is highly dependent on the time of sampling. Nevertheless, the present work is only the second published analysis of asymptomatic EBV acquisition in adulthood and the only one using a wide range of immunological tools to allow direct comparison with individuals of a similar age who developed acute IM.

The first of several important questions concerned the viral loads in individuals with asymptomatic infection. Here the message was clear. Three individuals, AS1, AS2, and AS5, with an EBV antibody profile equivalent to that seen in patients during the acute phase of IM (anti-VCA IgM^+^, anti-VCA IgG^+^ or IgG^−^, anti-EBNA1 IgG^−^) had circulating EBV genome loads well within the range of those in patients during the acute phase of IM, whereas a fourth individual, AS6 (anti-VCA IgM^+^, anti-VCA IgG^+^, anti-EBNA1 IgG^−^), had a relatively low load, although it may be that we had missed an earlier peak of infection in this particular case. The other two individuals, AS3 and AS4, had loads that were just below the range of those found in patients with IM; however, the fact that neither individual had yet mounted a detectable anti-VCA IgM response suggests that they may have been caught relatively early in the course of infection, with the EBV load in the blood possibly still rising. These findings reinforce those in the earlier report of asymptomatic adult infections, where two of three anti-VCA IgM^+^ individuals tested had elevated viral loads in PBMCs similar to those in patients with IM (37). Together the two studies make it clear that, while the severity of symptoms in patients during the acute phase of IM is reported to correlate directly with the viral load in the blood (5), the viral load *per se* cannot be the driver of symptoms since most individuals with asymptomatic infections identified to date display similarly high loads.

We stress that these elevated EBV loads refer to cell-associated virus genomes and almost certainly reflect the high frequencies of latently infected cells present within the circulating B cell pool. We could not prove this directly in the present work because the limited number of blood samples from AS individuals precluded isolation of the B cell fraction. However, many previous studies in subjects with high PBMC loads, whether they were IM patients, immunocompromised transplant recipients, or healthy individuals, have shown that EBV is selectively carried in B cells and as a latent, not lytic, infection (10, 11, 41, 42). This speaks against virus replication as a major contributor either to cell-associated viral loads or, through virus shedding, to cell-free viral DNA in plasma. In the latter context, even in the most acute cases of IM with huge B cell loads, viral genomes are only transiently detectable in plasma and are detectable at very low levels (12, 43). At the same time, it is important to recognize that studies of PBMCs and/or circulating B cells tell us nothing about the oropharyngeal infection, in particular, about the level of infectious virus shedding in the throat. We did not take throat washings in the present work. Recently, however, two prospective studies of EBV-seronegative students sampled both sites at regular intervals and found that, in those individuals who subsequently developed IM, virus shedding did not become detectable until very close to or at the time that symptoms appeared (5, 6), suggesting that there is little oropharyngeal replication occurring in the long prodromal phase. This reinforces the idea that oropharyngeal B cells are the first target of orally transmitted virus and that foci of lytic replication in permissive epithelium are dependent upon seeding from locally infected B cells. Interestingly, in contrast to the viral load in the blood, the levels of virus replication in the throats of IM patients bear no relationship to the severity of symptoms (5); indeed, such discordance is also apparent after the acute phase of IM, with oral shedding typically remaining high long after the resolution of symptoms (5, 12, 17).

We next examined aspects of the cell-mediated response in asymptomatic infection. In their earlier study, Silins et al. (37) found no evidence that their subjects had raised lymphocyte counts at the time of infection; however, they were unable to look at the key features affecting the blood picture in patients with IM, namely, CD8^+^ T cell activation and the induction of a massive EBV antigen-specific CD8^+^ T cell response (15, 16). In the present work, we found that three of our five prospectively studied subjects had an ongoing virus-specific CD8 response at the time that EBV was first detected, and in two of those individuals (AS1 and AS2), this was coincident with significant levels of activation in the CD8^+^ T cell pool; however, overall CD8^+^ T cell numbers never rose to the levels seen in patients during the acute phase of IM. In the case of AS1, while the total lymphocyte counts were not raised at the time of infection, the percentage of CD8^+^ T cells was higher than that in subsequent blood samples, and almost 40% of those CD8^+^ T cells were activated. Furthermore, tetramer staining showed that about 15% of that activation could be explained by the response to one EBV lytic cycle epitope, while a blood sample taken 6 months later revealed a delayed response to two further epitopes. In the case of AS2, a somewhat larger CD8^+^ T cell expansion sufficient to cause a detectable (but still <2-fold) increase in total lymphocyte counts was observed. Some 45% of the expanded CD8 population was activated, and the activated population contained CD8^+^ T cell responses to three EBV lytic epitopes. Both AS1 and AS2 therefore showed elements of a CD8^+^ T cell response like that in patients during the acute phase of IM but in a less exaggerated form. How much of the overall CD8^+^ T cell activation can be ascribed to EBV-specific cells in such cases remains a moot point. Some of this may reflect bystander activation, as acute EBV infection has been shown to induce an activated phenotype (though not proliferation) in preexisting CD8^+^ T cell memory to unrelated viruses (44). However, we anticipate that the EBV-specific response is a major contributor because our tetramer assays, focusing on a small number of epitopes restricted through just one or two HLA alleles, clearly underestimated the true size of that response.

In contrast to the cases described above, AS3 and ASS4 showed no evidence of any lymphocytosis or general CD8^+^ T cell expansion/activation in the first virus-positive blood sample. However, since both individuals may have been sampled relatively early in the course of infection and were not resampled until much later, it may be that a subsequent expansion occurred and was missed. This remains a possibility, but, at least in the case of AS4, an EBV-specific CD8^+^ T cell response was already detectable without disturbance of the CD8^+^ T pool as a whole. AS3 and AS4 may therefore represent cases with positions at the other end of the asymptomatic spectrum, i.e., cases where control of the infection occurs without an obvious impact on the CD8 pool as a whole. Most interesting was the case of AS5, who, despite already sustaining high EBV loads in the blood, showed no lymphocytosis, no CD8 expansion/activation, and no detectable EBV-specific CD8^+^ response either during the acute phase of infection or in a blood sample taken 3 months later. It was not until 16 months after the time of collection of the first blood sample that an EBV-specific CD8^+^ T cell response was detected, at which time cells reactive to three lytic epitopes were present, and some still had a phenotype suggesting relatively recent activation. Later blood samples detected the same CD8^+^ T cell reactivities in memory, and this was joined in the blood sample collected 26 months after the time of collection of the first blood sample by a recently activated response to a subdominant latent epitope. Note that there is a parallel between this long-delayed T cell response to infection and the same individual's unusually slow IgG (i.e., T cell-dependent) antibody response to the virus; thus, AS5 was still anti-VCA IgG^−^ 3 months after the primary infection and still anti-EBNA1 IgG^−^ after 16 months.

Several lines of evidence both from IM patients (5, 45) and from children with primary immune deficiencies (46, 47) suggest that, besides T cells, the NK cell system has a role to play in restraining EBV infection. As to the mechanism of control, early interest came from the finding that CD56^bright^ NK cells (the dominant NK cell subset in lymphoid tissues) have the potential to delay EBV-induced B cell transformation *in vitro* by producing IFN-γ (48). However, more recent work studying EBV infection in the humanized mouse model suggests that NK cells target lytic rather than growth-transforming latent infections (49). This accords with *in vitro* evidence that latently infected B cells become susceptible to NK cell recognition and killing only upon entry into the lytic cycle (50). Interestingly, the CD56^dim^ NKG2A^+^ KIR^−^ subset that best mediates such lytic cycle recognition *in vitro* is also the subset that is preferentially expanded in the blood of IM patients (19). This has prompted the hypothesis that NK cells play an important role early in the host response to primary EBV infection by controlling lytic virus replication, thereby limiting the amount of infectious virus entering the B cell system and also reducing the yield of lytic antigens that are the main drivers of the primary CD8^+^ T cell response (49). By this argument, since IM is a disease associated with high viral loads and an exaggerated T cell response, it may arise as a result of impaired NK cell control; conversely, asymptomatic infection would be associated with efficient NK cell responses. In the present study of asymptomatically infected individuals, we therefore extended our analysis to include the circulating NK cell population and its subsets. One possibility was that, in comparison to the small expansion in NK cell numbers and slight increase in CD56^dim^ NKG2A^+^ KIR^−^ representation reported in IM patients (19), these indicators of an active NK cell response would be magnified in individuals with asymptomatic infection. Using protocols that confirmed the reported NK cell changes in the blood of IM patients, we found (in at least four of the five individuals studied) no clear evidence for expansion either of total NK cells or of the CD56^dim^ NKG2A^+^ KIR^−^ subset. The one partial exception was AS3 (one of the two cases possibly caught early postinfection), in whom the percentage representation of total NK cells was increased in the lymphocyte population at the time that EBV was first detected; however, this was not accompanied by any sign of NK cell activation or a subset shift. Taken overall, our findings suggest that, contrary to the prediction made above, asymptomatic infection is not characterized by an NK cell response that is magnified compared to that seen in IM patients. However, we would add two caveats to this conclusion. First, it is possible that the key NK cell responses are only transiently reflected in the blood and were simply missed because of the irregular monitoring of our AS cases, especially in those individuals who (unlike patients during the acute phase of IM) had not yet become anti-VCA IgM^+^. Second, the composition of NK populations in the blood does not necessarily reflect what is occurring at the presumed site of NK cell action, in oropharyngeal tissues.

Little is known about EBV's interaction with other innate immune cell lineages. In this regard the potential importance of myeloid cells and, in particular, DCs is interesting because the virus genome encodes a unique lytic cycle protein, BARF1, which blocks colony-stimulating factor 1 (CSF-1), a cytokine promoting myeloid cell proliferation/function (51). Moreover, in the macaque model with the EBV-related rhesus lymphocryptovirus, deletion of the rhesus lymphocryptovirus BARF1 coding sequence (hence, the loss of BARF1's immune-evasive effects) led to lower viral loads during the acute stage of infection (52), implying that the early events of virus infection *in vivo* are indeed subject to immune pressure from the myeloid lineage. Reasoning that the recently described loss of pDCs and mDCs from the blood of patients during the acute phase of IM (20, 22) might reflect active mobilization of such an innate response toward the site of infection, as observed in other viral systems (53, 54), we asked whether the same fall in the number of circulating DCs was also detectable in asymptomatic cases. Interestingly, both AS1 and AS2, the two individuals with asymptomatic infections that had viral loads and T cell response kinetics most like those in patients during the acute phase of IM, did indeed show lower levels of pDC and mDC subset representation (as a percentage of all PBMCs) in the blood sample collected at the time of primary infection compared to those seen in blood samples collected at later times. In contrast, AS5 showed no significant change, even though AS5 had circulating EBV genome loads as high as those in patients AS1 and AS2 this is at least consistent with the idea that, in this individual, not only the adaptive immune response but also parts of the innate immune response were delayed.

The work was then extended to another reported feature of acute IM: the increase in the levels of several proinflammatory cytokines/chemokines in plasma (23–26). Screening for a range of these candidates, we found that the levels of many were elevated in some but not all samples from patients during the acute phase of IM; this likely reflects the transient nature of some cytokine/chemokine elevations in patients during the acute phase of IM, with levels rising and falling at particular times even within the disease course itself. In contrast, we identified three markers that appeared to be less susceptible to such variability, being consistently increased across our panel of samples from patients during the acute phase of IM: these were IL-18, known to induce IFN-γ production (55), and IP-10 and MIG, both of which are induced by IFN-γ (56). Going on to study these markers in the individuals with asymptomatic infections, we found some interesting changes, though the magnitude of change was always less marked than that in patients during the acute phase of IM. Thus, the blood samples collected from AS1 and AS2 at the time of acute infection showed slight rises in IL-18 levels over those at the baseline and larger increases in MIG and (for AS1) also in IP-10 levels. In contrast, both AS3 and AS4 showed no such effect, possibly because they were sampled relatively early in the course of infection. More interestingly, cytokine levels were also not increased in the blood sample from AS5 with a high viral load collected at the time of acute infection; however, the levels of both MIG and IP-10 were significantly elevated (compared to earlier and later levels) in the blood sample taken at 16 months, just after the peak of the virus-specific CD8^+^ T cell response. This chimes with recent work on the changing transcriptional profile of PBMCs over the course of IM, where upregulation of IFN-γ pathway genes strongly correlated with CD8^+^ T cell expansion rather than other parameters (57). We provisionally conclude that, for those cytokines/chemokines that are most markedly elevated during primary EBV infection, the cytokine storm is not a product of the virus infection itself but of the exaggerated immune response to infection.

In summary, the present study helps to resolve some of the questions that surround asymptomatic primary infection, notably, with respect to the latent viral load in the blood, which can be as high as that in patients during the acute phase of IM, and the cell-mediated immune response, which may be qualitatively similar to that in patients during the acute phase of IM but never matches its size. However, our observations also emphasize the need for further, more intense, studies and highlight the challenges that such studies face. In particular, one does not know the point at which samples from individuals with asymptomatic infection are directly comparable to those from patients during the acute phase of IM because the kinetics of infection (virus acquisition, oropharyngeal replication, entry into the circulating B cell pool) may be different in the two situations. Furthermore, the present results, albeit from a limited study, strongly suggest that asymptomatic infections themselves do not follow a uniform kinetic path. For example, AS1 was similar to IM patients in mounting a prompt CD8^+^ T cell response to primary infection followed by a T cell-dependent IgG antibody response to both VCA and EBNA1; in contrast, in AS5, who was caught with an equally high viral load at the time of primary infection, the responses were delayed by at least several months. Intriguingly, these response kinetics did not correlate with the anticipated changes in the latent viral load; thus, despite mounting a prompt response, AS1 still had a high viral load in the blood at 6 months postinfection (though it fell to normal levels after that time), whereas the viral load in AS5 had fallen significantly within 3 months in the apparent absence of any T cell-mediated response.

Interpretation of these findings is difficult without a proper understanding of the early events that occur in the oropharynx following oral transmission of the virus (8, 9, 17, 20). Important prospective studies identifying subjects in the 5- to 7-week prodromal phase of IM have shown that high viral loads in both throat washing and blood samples do not appear until about the time of onset of symptoms (20); the authors suggest that a slow smoldering infection of the local B cell system may take weeks before expansion to a magnitude that drives an immune hyperreaction (20). With respect to that hyperreaction, evidence both from X-linked lymphoproliferative disease studies (58–60) and from the humanized mouse model (49) strongly suggest that lytically infected B cells, as opposed to the mucosal epithelium, are the main drivers of both the NK and highly expanded CD8^+^ T cell responses seen in patients with IM. The balance between lytic and latent B cell infection is finely drawn, and it seems possible that, if the local environment within the oropharynx favors latency, then the general B cell system might be colonized without putting the NK and T cell systems on high alert. The inference is that, compared to patients during the acute phase of IM, asymptomatic infections would be characterized by lower oropharyngeal replication of the incoming virus and, hence, a lower lytic antigen load. More broadly, perhaps the most important lesson from this work is that not all asymptomatic infections necessarily follow the same course; while some may be like those seen in patients during the acute phase of IM in the timing and quality of the immune responses, others may be quite different and ultimately reveal novel mechanisms of host control.

## MATERIALS AND METHODS

### Study cohort.

Initial blood samples were collected from a total of 448 medical students recruited either between October and December 2007 or between October and December 2009 when entering their degree course. Subject to consent, follow-up samples were collected in year 2 (February or March), year 3 (December or January), and year 5 (February to May) of their studies. Additional samples were requested from particular individuals if subsequent assays revealed recent EBV infection. This study was approved by the West Midlands (Edgbaston) Research Ethics Committee (REC reference 07/H1208/40), and participants gave written informed consent in accordance with the Declaration of Helsinki.

### Sample collection and preparation.

PBMCs were isolated from a 20-ml heparinized blood sample by Ficoll/Hypaque density gradient centrifugation. In each case, an aliquot of 1 × 10^6^ to 2 × 10^6^ PBMCs was frozen for later DNA extraction to determine the HLA genotype and EBV genome loads, while the remaining PBMCs were cryopreserved as duplicate ampoules in RPMI 1640 with 20% fetal calf serum and 10% dimethyl sulfoxide. Plasma was frozen to identify EBV and CMV serostatus. These samples were studied in parallel with cryopreserved PBMC and frozen plasma samples collected prospectively from IM patients during the acute phase and at later times after the resolution of symptoms (16, 61). Full blood counts to determine lymphocyte numbers were performed at the Hematology Laboratory, Queen Elizabeth Hospital, Birmingham, United Kingdom.

### Determining EBV and CMV serostatus.

Serial dilutions of plasma were assayed for the presence of IgM and IgG antibodies to EBV VCA by indirect immunofluorescence as described previously (62); as VCA-positive and -negative target cells, the Akata-BL and EBV-loss Akata-BL lines were used in the IgM assay, and the P3HR1 and BJAB lines were used in the IgG assay. The titers of IgG antibodies to EBNA1 were determined using a commercially available diagnostic enzyme-linked immunosorbent assay according to the manufacturer's instructions (Diamedix, Miami, FL). CMV serostatus was determined as described previously (63).

### Determining EBV genome load in PBMCs.

Genomic DNA was isolated from PBMC pellets using a GenElute blood genomic DNA kit (Sigma-Aldrich) according to the manufacturer's instructions. DNA was eluted into 80 μl buffer, quantified using a NanoDrop spectrophotometer, and stored at −20°C. A multiplex quantitative PCR assay which simultaneously amplifies EBV BALF5 (DNA polymerase) and cellular beta-2-microglobulin sequences was used to determine the EBV genome load (64); four replicate samples of 500 ng DNA (each equivalent to 1.5 × 10^5^ cells) were assayed for each PBMC preparation. All standards and samples were tested in triplicate, and the data were analyzed using SDS (v1.4) software.

### Flow cytometric analysis of EBV-specific and total T cell populations.

Tetramers were used to identify and analyze the surface marker phenotype of EBV-specific CD8^+^ T cells. In addition to CD3 and CD8, cell surface CD45RA and CCR7 were used to identify the naive (CD45RA^+^ CCR7^+^), central memory (CD45RA^−^ CCR7^+^), effector memory (CD45RA^−^ CCR7^−^), and revertant memory (CD45RA^+^ CCR7^−^) T cell subsets. Staining for cell surface HLA-DR and CD38 and for intracellular Bcl2 was used to identify activated (HLA^−^ DR^+^ CD38^+^ Bcl2^low^) T cells. Tetramer staining identified CD8^+^ T cells specific for the following epitopes derived from individual EBV lytic or latent cycle proteins (see below): YVLDHLIVV (lytic, BRLF1), GLCTLVAML (lytic, BMLF1), LLIEGIFFI (lytic, BaRF1), or CLGGLLTMV (latent, LMP2), which are restricted through HLA-A*0201, or RPRATWIQEL (lytic, BaRF1), TPSVSSSISSL (lytic, BFRF3), or RPPIFIRRL (latent, EBNA3A) (14), which are restricted through HLA-B*0702. Epitopes are designated by their initial three residues (underlined).

Tetramer and antibody staining of cryopreserved PBMCs was performed as follows. Cells were thawed and stained with LIVE/DEAD fixable Aqua Dead cell stain (Molecular Probes, Thermo Fisher Scientific) for 15 min at room temperature, washed, and stained with tetramer-phycoerythrin (PE) for 15 min at 37°C. Following two further washes, surface staining with the following was performed in Brilliant stain buffer (BD Biosciences) for 30 min at 4°C: anti-CD14-Pacific Green (clone SJ25-C1) and anti-CD19-Pacific Green (clone TüK4) (both from Molecular Probes, Thermo Fisher Scientific); anti-CD3-BV786 (clone SK7), anti-CD8-allophycocyanin (APC)-H7 (clone SK1), anti-CD45RA-BV605 (clone HI100), and anti-CD45RO-BV711 (clone UCHL1) (all from BD Biosciences); anti-CD38-peridinin chlorophyll protein (PerCP)-Cy5.5 (clone HIT2) and anti-HLA-DR-APC (clone L243) (both from BioLegend); and anti-CCR7-fluorescein isothiocyanate (clone 150503; R&D Systems). Following fixation and permeabilization using a Cytofix/Cytoperm kit (BD Biosciences), intracellular staining with anti-Ki67-BV421 (clone B56) and anti-Bcl-2-PE-CF594 (clone 563601) (both from BD Biosciences) was performed in Brilliant stain and Perm/Wash buffer (both from BD Biosciences) for 30 min at 4°C.

Flow cytometry data were acquired with an LSR Fortessa X20 analyzer (BD Biosciences) with standard filter sets. Data were analyzed using Kaluza (v1.3) software (Beckman Coulter), and figures were created using FlowJo software (TreeStar). CD3^+^ T cells were gated on within the single, viable, CD14^−^ CD19^−^ lymphocyte population, before CD8^+^ T cells and CD8^+^ tetramer-positive cells were selected for further analysis of surface and/or intracellular marker expression.

### Flow cytometry-based analysis of NK cells and DCs.

PBMCs were thawed and washed twice in R10 medium (RPMI 1640 [Sigma-Aldrich] containing 10% fetal bovine serum, 50 IU/ml penicillin, and 50 μg/ml streptomycin). Cells were counted and resuspended in R10 medium at a concentration of 2 × 10^6^ cells/ml. NK cells were identified by staining 2 × 10^5^ PBMCs with LIVE/DEAD violet amine dye (Life Technologies), anti-CD19-Pacific Blue (PB) (clone LT19; AbD Serotec), and anti-CD3-PB (clone SP34-2), anti-CD14-PB (clone M5E2), and anti-CD56-PE-Cy7 (clone B159) (all from BD Bioscience) and gating on live, lineage-negative lymphocytes expressing CD56 (Fig. 6A). Receptor expression on CD56^+^ NK cells and subsets thereof was evaluated using the following antibodies: anti-CD158a/h-biotin (KIR2DL1/S1 clone 11PB6; Miltenyi), anti-CD158b (KIR2DL2/L3/S2 clone CH-L; BD Bioscience), anti-CD158e1/e2 (KIR3DL1/S1 clone Z27) and anti-NKG2A-PE (clone Z199) (both from Beckman Coulter), and anti-HLA-DR-APC (clone L243) (BioLegend). Streptavidin-Pacific Orange (Life Technologies) was used to detect anti-CD158a/h-biotin. Intracellular expression of Ki67 and Bcl2 was assayed as described above for T cells.

DC subsets were identified by staining 5 × 10^5^ PBMCs with LIVE/DEAD violet amine dye (Life Technologies); anti-HLA-DR-PerCP (clone L243; BD Bioscience); anti-CD123-PE-Cy7 (clone 7G3; eBioscience); anti-CD11c-APC-H7 (custom conjugate; ReaMetrix); anti-CD3 (clone SP34-2), anti-CD14 PB (clone M5E2), and anti-CD16 PB (clone 3G8) (all from BD Bioscience); anti-CD19-PB (clone LT19) and anti-CD20 (clone 2H7) (both from AbD Serotec); and anti-CD56-PB (custom conjugate; ReaMetrix); gating on live, lineage-negative mononuclear cells expressing HLA-DR^+^ cells; and then identifying pDC and mDC subsets within this population on the basis of the differential expression of CD123 and CD11c (Fig. 7A).

Flow cytometry data were acquired with a Cyan ADP analyzer (Beckman Coulter) with standard filter sets. Data were analyzed using FlowJo software (v8.8.6; TreeStar).

### Plasma cytokine assays.

Plasma levels of IFN-β, IFN-γ, IL-1β, IL-6, IL-10, IL-12, IL-18, TNF-α, MIG/CXCL9, and IP-10/CXCL10 were determined by a Luminex assay as described previously (65).

### Statistical tests.

All graphical data and statistical analyses were generated using Prism software (v5; GraphPad Software Inc., San Diego, CA). The statistical significance of differences between values measured at the first viremic time point and time points after 6 months in each group of subjects was determined using a Mann-Whitney nonparametric test; *P* values of <0.05 were considered significant.
